# Diversity of *Culicoides* in the middle belt of Ghana with Implications on the transmission of *Mansonella perstans*; a molecular approach

**DOI:** 10.1186/s13071-024-06179-8

**Published:** 2024-03-12

**Authors:** Linda Batsa Debrah, Joseph F. Arthur, Augustine Yeboah, Dorcas O. Owusu, Ernest Adankwah, Isaac Acheampong, Difery Minadzi, Millicent Lamptey, Vera Serwaa Opoku, Wilfred Aniagyei, Monika M. Vivekanandan, Mohammed K. Abass, Amidu Gawusu, Samuel Wanji, Alexander Y. Debrah, Marc Jacobsen, Richard O. Phillips

**Affiliations:** 1 Kumasi Centre for Collaborative Research in Tropical Medicine (KCCR), Kumasi, Ghana; 2 Department of Medical Diagnostics, College of Health Sciences, Kwame Nkrumah University of Science and Technology (KNUST), Kumasi, Ghana; 3 Agogo Presbyterian Hospital, Agogo, Ghana; 4 Sene West Health Directorate, Kwame Danso, Ghana; 5 Department of Microbiology and Parasitology, University of Buea, Buea, Cameroon; 6 Department of General Pediatrics, Neonatology and Pediatric Cardiology, Medical Faculty, University Hospital Duesseldorf, Heinrich-Heine University, 40225 Duesseldorf, Germany; 7 School of Medicine and Dentistry, College of Health Sciences, KNUST, Kumasi, Ghana; 8 Department of Clinical Microbiology, School of Medicine and Dentistry, Kwame Nkrumah University of Science and Technology (KNUST), Kumasi, Ghana

**Keywords:** *Culicoides species*, *Culicoides grahamii*, *Mansonella perstans*, Microfilariae

## Abstract

**Background:**

*Culicoides*, also known as biting midges, carry pathogens which include *Mansonella perstans*. *Mansonella perstans* is a nematode parasite implicated in a number of disease outcomes. Even though a high prevalence of about 75% *M. perstans* infection has been recorded in some communities in the middle belt of Ghana, and a wide diversity of *Culicoides* species has been identified, the exact *Culicoides* species transmitting *M. perstans* in Ghana has not yet been deciphered. This study therefore aimed at assessing the species diversity of *Culicoides* and their role in the transmission of *M. perstans* in the middle belt of Ghana.

**Methods:**

*Culicoides* species were sampled from 11 communities in the Asante-Akim North and Sene West districts in the middle belt of Ghana. Centre for Disease Control (CDC) UV light traps, as well as human bait (i.e. human landing catch and engorged catch) methods were used to assess the species abundance and diversity of *Culicoides* in the study communities in the wet and dry season. A colorimetric Loop-Mediated Isothermal Amplification (LAMP) assay was performed to assess the vector competence of the various *Culicoides* species.

**Results:**

A total of 4810 *Culicoides* from 6 species were sampled. These included *Culicoides inornatipennis, C. milnei, C. schultzei, C. grahamii, C. neavei*, and *C. imicola. Culicoides imicola* was the most abundant species (56%) followed by *C. grahamii* (16%). Light traps sampled the most diverse species (6 species). Human landing catch and engorged catch methods identified three anthropophilic species, *C. grahamii, C. milnei*, and *C. inornatipennis*, with *C. grahamii* being the most anthropophilic with a peak biting time between the hours of 5 p.m. to 6 p.m. Generally, there was relatively higher species abundance in the wet than dry season. LAMP assay identified *C. grahamii* as the potential vector for *M. perstans* transmission in the middle belt of Ghana.

**Conclusions:**

For the first time, we have demonstrated that *C. grahamii* is the potential competent vector for *M. perstans* transmission in the middle belt of Ghana. It is more abundant in the rainy season and has a peak biting time between the hours of 5 and 6 p.m.

**Graphical Abstract:**

**Figure:**
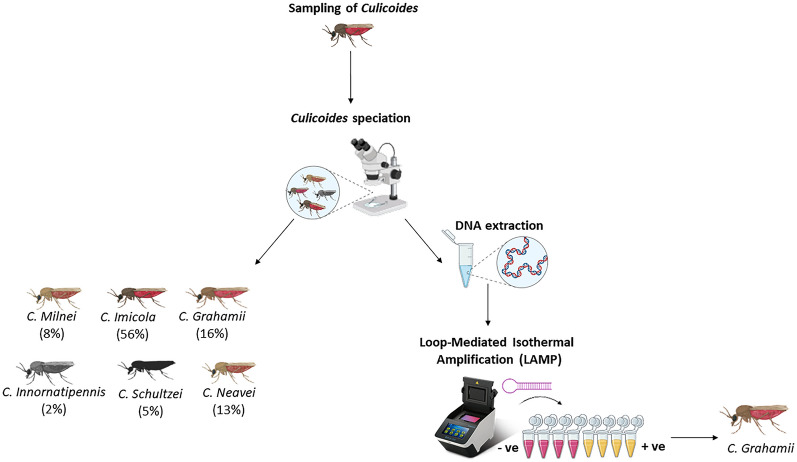

**Supplementary Information:**

The online version contains supplementary material available at 10.1186/s13071-024-06179-8.

## Background

Biting midges of the genus *Culicoides*, family Ceratopogonidae, have a near worldwide distribution with over 1400 species described [1]. These vectors are small, stout hematophagous flies with a distinctive pattern of dotted wings. They are a biting nuisance to people, domestic animals, and wild animals in regions where they are prevalent. Their bites can occasionally cause allergic skin reactions, which result in urticaria in some individuals [2].

Biting midges have been incriminated as the vectors of parasites as well as multiple arboviruses of veterinary and public health importance [3] such as Schmallenberg virus and Bluetongue virus in ruminants [4]. Their occurrence, abundance, and diversity vary among different ecological zones and seasons. The lifestyle of *Culicoides* is characterized by complete metamorphosis from the egg through larval and pupal stages until the adult stage [5]. The presence of moisture-rich habitats is essential for the development of pupal and larval stages, as such *Culicoides* are mostly associated with aquatic and semi-aquatic habitats, e.g. mud, marshes, and ponds. Characteristically, they can be found in areas where there are many plantain farms. The decaying leaves and stems of plantain serve as a good breeding ground for these flies. The presence of livestock also plays a crucial role in the distribution of *Culicoides* [6].

*Culicoides* spp. are vectors for transmitting the nematode parasite *Mansonella perstans* [7]. The life cycle of *M. perstans* is similar to that of other nematode parasites such as *Onchocerca volvulus*, *Loa loa*, and *Wuchereria bancrofti*, where humans are the definitive host [2, 5].

About 114 million people in Africa are estimated to be infected with *M. perstans*, and over 580 million people globally are considered to be at risk [7]. Despite the wide distribution and high prevalence of *M. perstans* infection, it is not officially regarded as one of the Neglected Tropical Diseases (NTDs) and has received minimal attention [2]. Unlike other human filarial infections that have well-defined clinical signs and symptoms, such as in *W. bancrofti* infection and the formation of subcutaneous nodules in *O. volvulus* infection, *M. perstans* infection does not present with any distinct or peculiar clinical signs and symptoms [2]. However, *M. perstans* infection has been shown to modulate the immune system resulting in complications in disease manifestations and recovery [2, 8].

Some *Culicoides* species are anthropophilic (attracted to humans) whereas others are not. Moreover, not all anthropophilic species are competent vectors of *M. perstans*. In East Africa, the taxonomy of *Culicoides* has been investigated by Khamala & Kettle, who identified 61 species that did not contribute to the transmission of *M. perstans* [9]. Different geographical locations have reported diverse *Culicoides* species but very few studies have identified vectors of *M. perstans* in endemic areas. *Culicoides milnei* is implicated in the transmission of *M. perstans* in the southwest region of Cameroon [10], and *C*. *grahamii* is confirmed as the vector in Congo [11]. However, the vector for *M. perstans* transmission in Ghana remains unknown even though high infection prevalence has been reported [12]

Previously, identification of *M. perstans* vectors was by detection of infective larvae in the female adult *Culicoides* upon dissection by microscopy. This requires great expertise as a result of the morphological similarities of different microfilariae species. Molecular (DNA-based) techniques, particularly PCR for filarial parasite detection in both the human host and vectors, are sensitive and specific, and useful in *M. perstans* epidemiological surveys [2]. A number of isothermal amplification methods targeting DNA have been developed, which offer appreciable advantages over PCR [13]. Of these, loop-mediated isothermal amplification (LAMP) is a widely adopted approach. Its ability to be conducted at a stable temperature, sensitivity and specificity, visual detection formats without the need for sophisticated equipment, and good performance, even using crude DNA, offers considerable advantages over PCR [14].

We carried out an entomological survey to assess the species diversity of *Culicoides*, established their role in the transmission of *M. perstans* in the middle belt of Ghana, and sought to identify the potential vector for its transmission using a loop-mediated isothermal amplification assay.

## Methods

### Study design

The study was conducted in 11 communities within the middle belt of Ghana. Seasonal collections of *Culicoides* were carried out from June 2020 to October 2020 for the wet season and November 2020 to April 2021 for the dry season. *Culicoides* were collected during the 1st week in each month. Centre for Disease Control (CDC) light traps were used to assess the species diversity of *Culicoides* present in the study communities. Human landing catch (HLC) was used to identify anthropophilic *Culicoides* species. Engorged *Culicoides* collected by drop trap using a known microfilaria-positive donor as a bait were used to assess vector competence. DNA was extracted from all *Culicoides* collected, and the LAMP assay was performed to assess vector competence of *M. perstans* transmission by *Culicoides*.

### Collection of adult *Culicoides* species using CDC miniature UV light traps

Here, each community was divided into four quadrant centers. A CDC New Standard Miniature light trap (John W. Hock Company, Gainesville, FL, USA), was mounted in each quadrant center, near human habitations. Collections were made overnight (6 p.m. to 6 a.m.) each sampling day. Light traps were mounted at the same spot in the wet and dry seasons. Attracted by UV light emitted by the trap, *Culicoides* species and other flies were trapped in a Petri dish containing 80% alcohol placed in the suspended trap. The trapped flies were transferred into labelled 50-ml Falcon tubes containing 80% alcohol and placed in a cold box for transportation to the laboratory for morphological identification. The number of each *Culicoides* species sampled was recorded after morphological identification.

### Human landing catches (HLC)

To determine anthropophilic *Culicoides* species, flies were collected using the human landing catch technique in all study communities. HLC was carried out in the evening from 4 to 7 p.m. each sampling day. Sampling was done by four well-trained collectors dressed in protective clothing against midges. They were positioned in four randomly selected houses and provided with torches to aid collection in darkness. Female *Culicoides* seeking blood meals were directly aspirated as soon as they landed on collectors and were then transferred into hourly labelled netted plastic cups and transported to the laboratory for morphological identification and further assessment. The number of *Culicoides* collected each hour was recorded to assess the peak biting time of the various anthropophilic *Culicoides* species.

### Collection of engorged *Culicoides* from known *M. perstans*-positive volunteer using a drop trap

To elucidate the role of anthropophilic species in *M. perstans* transmission (vector competence assessment), engorged catch method was deployed. The principle underlying this technique stems from the transmission cycle of *M. perstans*. This method involved an *M. perstans* microfilaremic donor who acted as bait. Collections were made from 6 p.m. to 6 a.m. each day for 4 consecutive nights. The *M. perstans* microfilaremic volunteer sat under a netting cage trap, with the net raised about 200 cm above the ground. Upon exposure, *Culicoides* were attracted, and cage netting was lowered after the volunteer was exposed for about 10 min to trap the attracted *Culicoides*. After about 10 min of lowering the cage (which is the estimated time for *Culicoides* to be fully engorged), the flies were aspirated from the net and blown into labelled 50 ml Falcon tubes and transported to the laboratory for morphological identification and further assessment.

Once collected, *Culicoides* were kept alive for 8 days before laboratory experiments.

### Morphological identification of *Culicoides* species

Morphological identification was done by examination of wing pigmentation pattern under a dissecting microscope. In cases where wing pigmentation was not enough, other morphological features such as genitalia, maxillary palps, and inter-ocular space were used [15, 16].

### *Culicoides* DNA extraction and quantification

After morphological identification, *Culicoides* were pooled into groups of 100 prior to DNA extraction. Each pool consisted of the same species of *Culicoides*. Machery Nagel Bioanalysis Nucleospin Tissue Kit was used in the extraction and purification of DNA from the *Culicoides*. Samples were completely homogenized using a MagNalyser. Afterwards, 180 µl of Lysis Buffer T1 and 25 µl of proteinase K were added to homogenize the samples and incubated at 56 °C overnight for pre-lysis; 200 µl of Lysis Buffer B3 was added and incubated at 70 °C for 10 min to achieve complete lysis of the chitinous exoskeleton and other proteins. Subsequently, 210 µl of ethanol was added to the lysate and thoroughly vortexed to adjust the DNA binding conditions and to precipitate the DNA. The resulting solution was pipetted into Nucleospin tissue columns placed in a 2-ml collection tube and centrifuged at 11,000 g for 1 min. Two washes with wash buffers BW and B5, respectively, were performed to get rid of unwanted dissolved cellular components, after which the spin column membranes were dried at 11,000 g for 1 min. The spin columns were placed in new 1.5-ml sterile Eppendorf tubes; 50 µl of Buffer BE (elution buffer) was added to the dried spin columns, incubated at room temperature for 1 min, and centrifuged at 11,000 g to elute DNA bound to the nucleospin tissue columns. Elution was repeated to achieve maximum yield. After extraction of *Culicoides* DNA, the concentration and purity of the DNA was measured using DeNovix NanoDrop.

### Detection of *M. perstans* infection in *Culicoides* species using colorimetric Mp419 LAMP assay.

The LAMP assay was carried out with a primer set as shown in Additional file 1: Table S1. Working solutions of 10× primer mixes were prepared from primer stock as described in Additional file 2: Table S2. The reaction was carried out in a total volume of 20 μl (18 μl of the reaction master mix and 2 μl of the DNA template) in polymerase chain reaction (PCR) micro-tubes (Additional file 3: Table S3). Amplifications were performed using an Applied Biosystems GeneAmp^®^ PCR System 9700 as all reactions were incubated at 63 °C (isothermal condition) for up to 40 min. A sample was considered positive for *M. perstans* DNA if an obvious colour change from pink to yellow was observed by two independent assessors, while for the negative samples there was no colour change in the phenol red colour.

## Results

A total of 4810 *Culicoides* comprising six different species (*C. imicola, C. grahamii, C. neavei, C. schultzei, C. inornatipennis*, and *C. milnei*) were collected in the 11 study communities. All Afrisere and Dukusen in the Asante-Akim North District recorded the highest abundance of *Culicoides* accounting for 17.2 and 13.7%, respectively, with Bebuso, recording the least *Culicoides* abundance (1.5%) (Table 1).

**Table 1:** Relative abundance of *Culicoides* species in study communities

District	Community	Number collected (%)
Sene-West	Drobe	659 (13.7)
	Lemu	439 (9.1)
	Kwame Danso	177 (3.7)
	Kyeamekrom	435 (9.0)
Asante-Akim North	Abutantri	689 (14.3)
	Afisere	826 (17.2)
	Dukusen	501 (10.4)
	Nhyiaeso	425 (8.8)
	Serebuoso	311 (6.5)
	Bebuso	70 (1.5)
	Anokye Beemu	278 (5.8)
Total (%)		4810 (100)

Of the 4810 *Culicoides*, 95% of the collections were from the light traps whereas < 1% was sampled using the drop trap. The light trap collected the highest diversity of *Culicoides* (6 species) compared to the HLC (2 species) and drop trap (3 species).

*Culicoides imicola* was the most abundant species (56.2%) and *C. inornatipennis* was the least (1.3%) (Table 2).

**Table 2:** *Culicoides* species collected by light traps, human landing catch (HLC), and engorged catch

*Culicoides* species	Method of collection			Number collected (%)
	Light trap	HLC	Engorged catch	
*C. inornatipennis*	35	16	0	51 (1.3)
*C. imicola*	2714	0	0	2714 (56.2)
*C. grahamii*	624	137	31	792 (16.5)
*C. milnei*	308	54	15	377 (7.8)
*C. neavei*	620	0	0	620 (12.8)
*C. schultzei*	256	0	0	256 (5.4)
Total	4557 (95)	207 (4.3)	46 (0.7)	4810 (100)

**Fig. 1:**
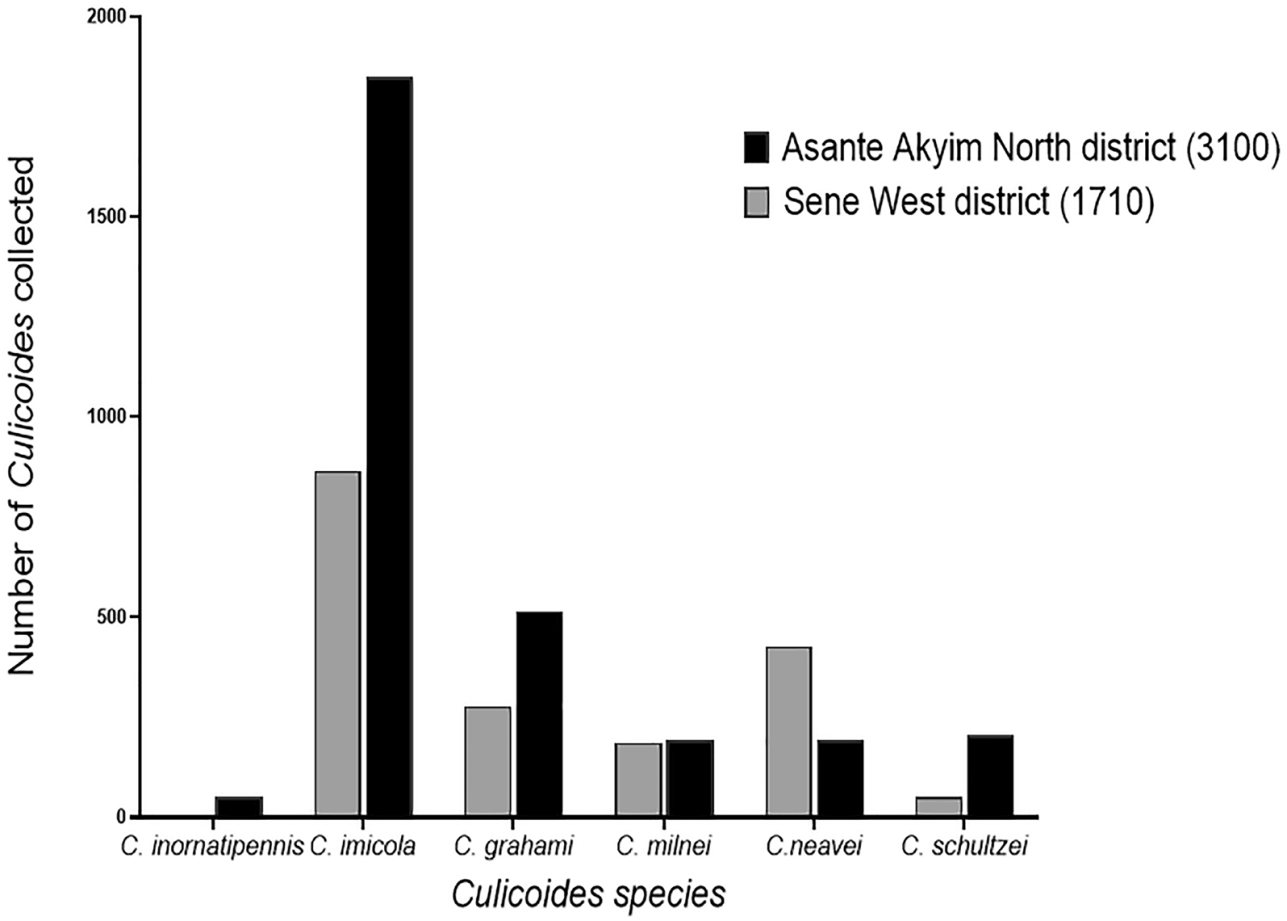
Abundance and diversity of *Culicoides* in the Asante Akim North and Sene West districts

**Fig. 2:**
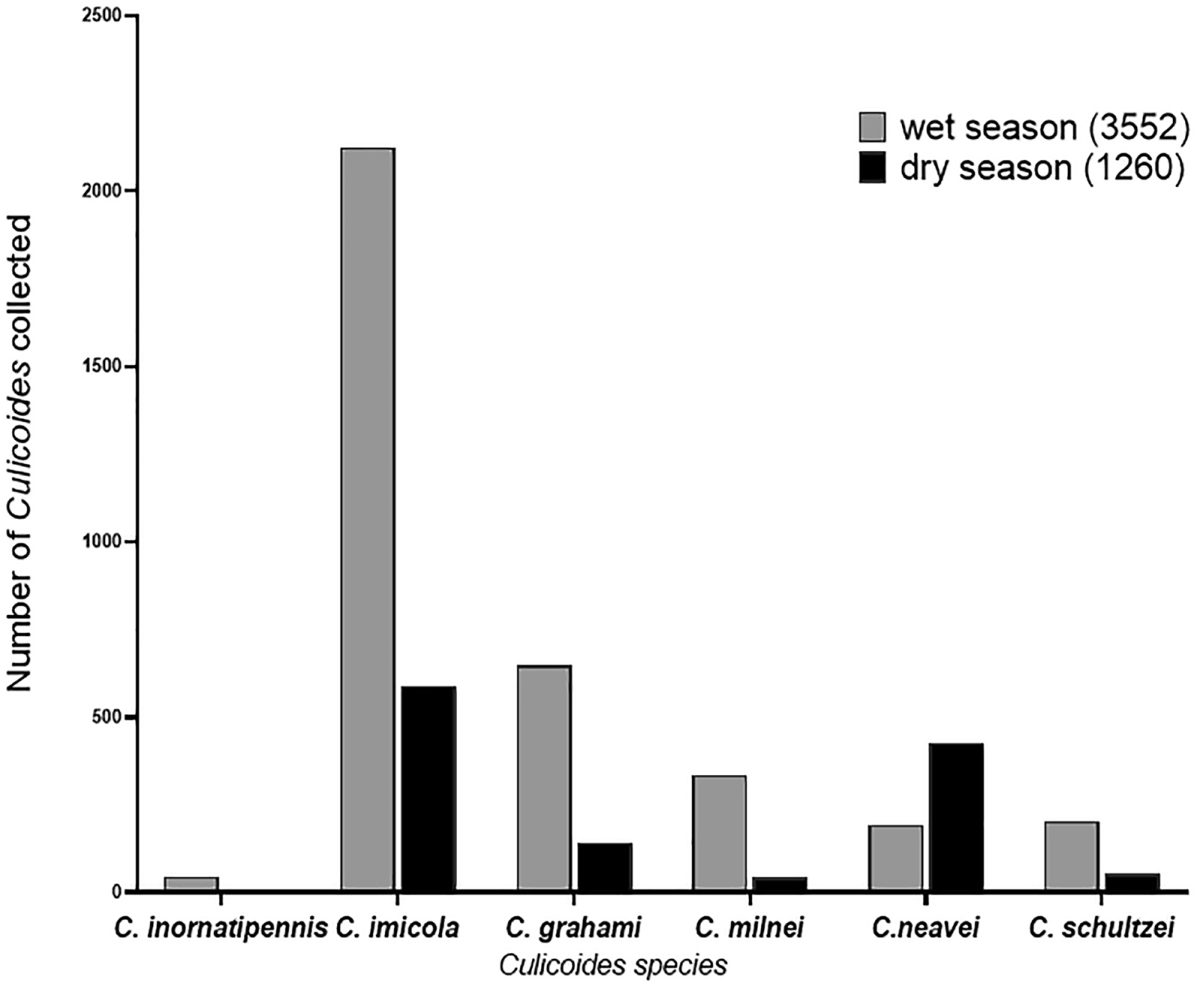
Seasonal variation of *Culicoides* abundance

The highest abundance of *Culicoides* was observed in the wet season except for *C. neavei*, which had higher abundance in the dry season (Fig. 2). Asante Akim North district had the highest number of *Culicoides* (6 species) and was the only district that recorded *C. inornatipennis* (Fig. 1).

**Fig. 3:**
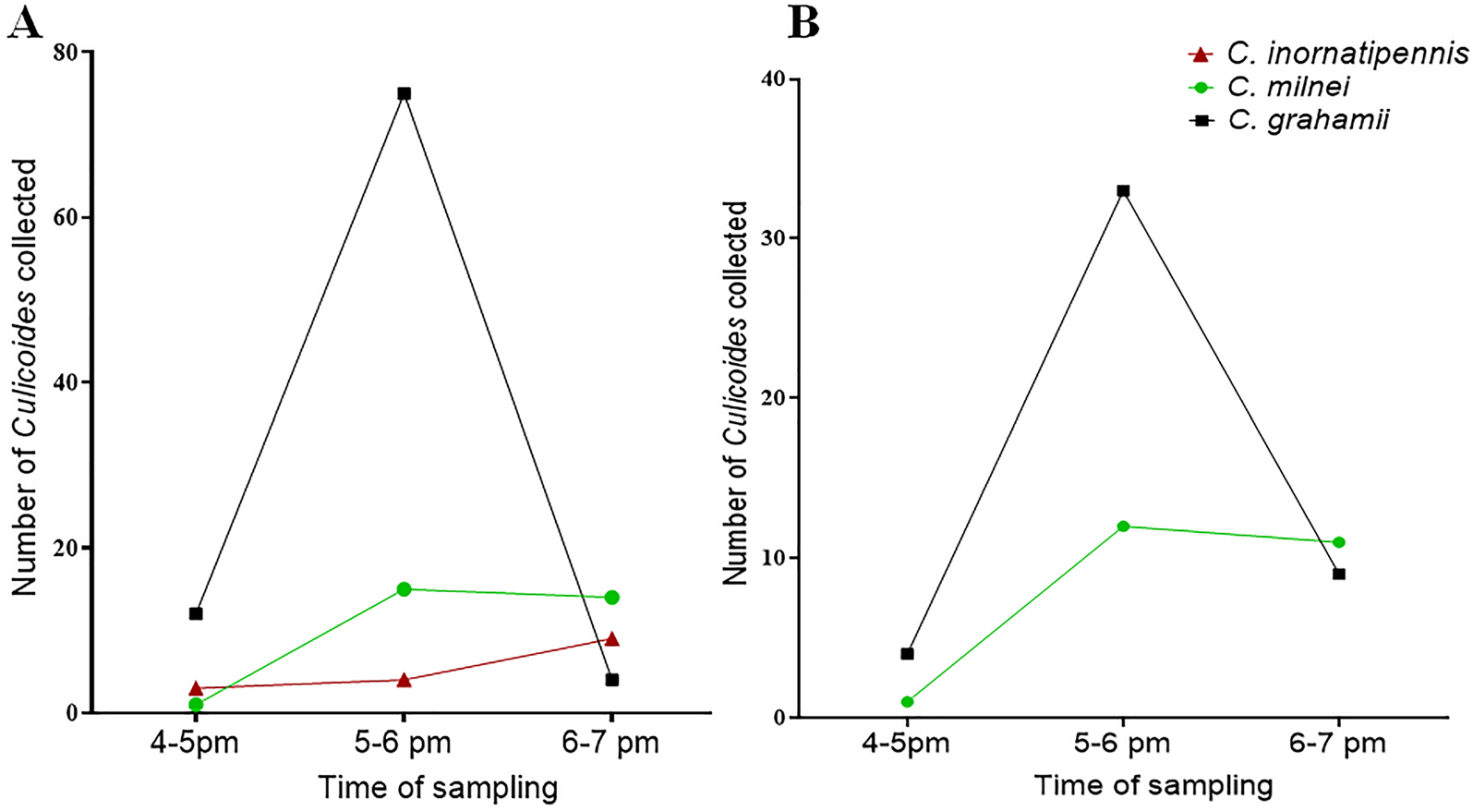
Biting patterns of anthropophilic *Culicoides* species in the Asante Akim North (**A**) and Sene West (**B**) districts

*Culicoides grahamii* was the most abundant anthropophilic species (66%). Both *C. grahamii* and *C. milnei* exhibited similarities in their biting pattern, showing a biting peak at 4–5 pm in the Sene West and Asante Akim North districts. Of the three anthropophilic species, *C. milnei* showed highest biting activity at 6 to 7 p.m. and the lowest biting activity at 4 to 5 p.m. (Fig. 3).

**Fig. 4:**
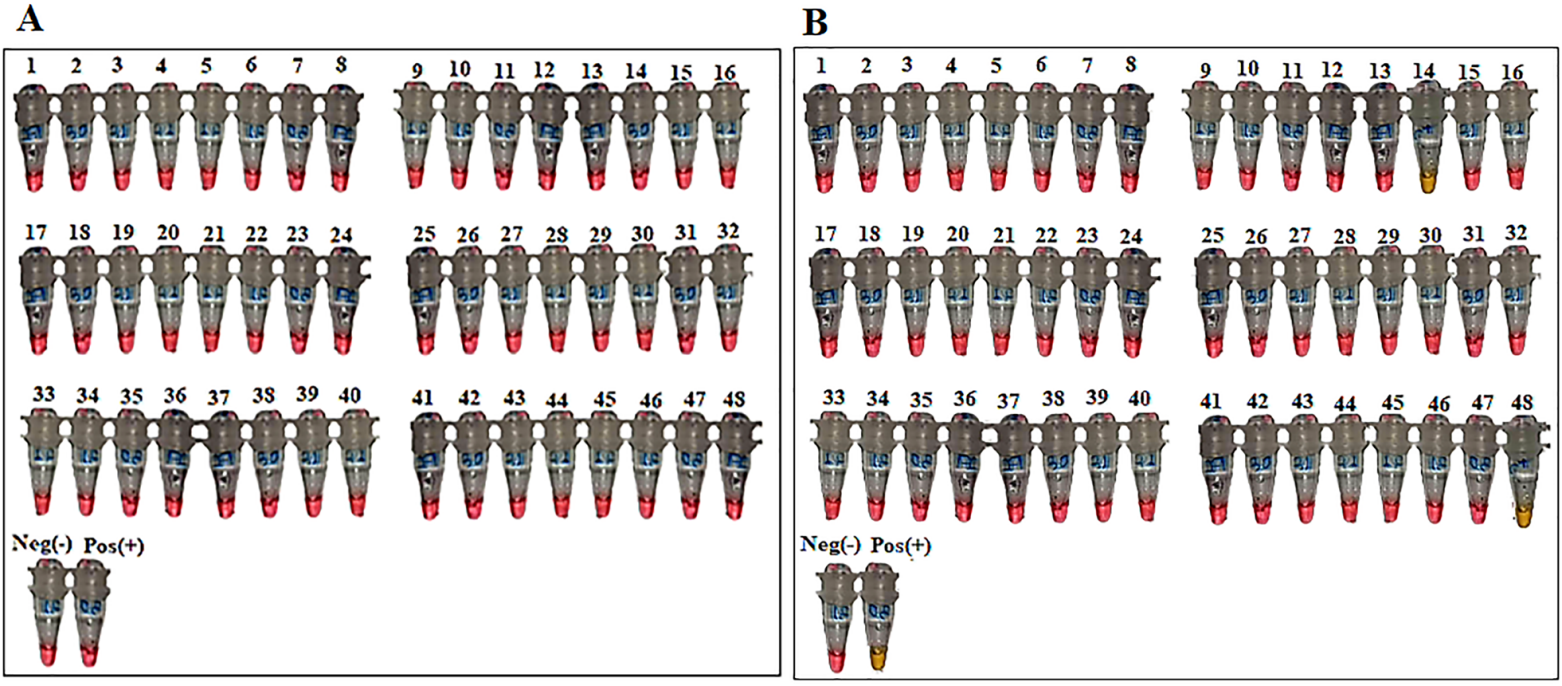
LAMP Assay reaction tubes before (**A**) and after reactions (**B**). *Culicoides neavei* light trap and HLC collections (1–6), *C. milnei* light trap and HLC collections (7–9), *C. grahamii* light trap and HLC collections (10–16), *C. schultzei* light trap collections (17–18), *C. imicola* light trap collections (19–45), *C. inornatipennis* light trap and HLC collections (46), *C. milnei* drop trap collections (47), *C. grahamii* drop trap collections (48), Pos ( +) positive control, Neg (−) negative control, pink colour indicates negative and yellow indicates positive

After the LAMP reaction, two pools of *C. grahamii* tested positive for *M. perstans* (showing a colour change from pink to yellow) (Fig. 4). These were observed in reaction tubes 14 (*C. grahamii* collected by light trap) and reaction tube 48 (*C. grahamii* collected by engorged catch) (Fig. 4).

## Discussion

Using colorimetric loop-mediated isothermal amplification assay [17], this study has successfully identified *C. grahamii* as the potential vector for *M. perstans* transmission in the middle belt of Ghana. A similar finding was made in the southwest region of Cameroon where *C. grahamii* was identified as a potential competent vector of *M. perstans* [18] even though *C. milnei* had been identified in earlier studies [10] to be the main vector in said region. This indicates that *C. grahamii* is susceptible to *M. perstans* invasion.

Six *Culicoides* species were identified in the surveyed communities in the current study, and all six *Culicoides* species were also identified by Debrah et al. [12], who were the first to comprehensively investigate *Culicoides* diversity and the burden of *M. perstans* infection in the middle belt of Ghana. Whereas Debrah et al.’s. [12] attempt to implicate specific *Culicoides* species as the vectors of *M. perstans* using microscopy was inconclusive, *C. grahamii* has proven to be effective in the carriage and transmission of *M. perstans* in the current study.

The engorged catch (using an overnight drop trap) as an experimental model to assess vector competence of anthropophilic *Culicoides* species was effective as only competent vectors are able to pick up *M. perstans* microfilariae from infected individuals and subsequently transmit it to another person through a blood meal. It was therefore not surprising that *C. grahamii* and *C. milnei*, being vectors identified in Cameroon, were the only species sampled by this method [10]. In addition to the drop trap, the detection of *M. perstans* infection in light trap collections (naturally infected *Culicoides)* is indicative of the Mp 419 LAMP assay’s ability to detect natural infection as well as experimental infection in *Culicoides*. This is important because it demonstrates the assay’s possible usage as a screening tool for *M. perstans* infection without having to go through experimental infection, which is time-consuming and possibly costly.

Several studies rely on the dissection of *Culicoides* post-infection with *M*. *perstans* [10, 19] to identify vectors with infective larvae (L3). This method requires high expertise as *Culicoides* species are dissected to identify *M. perstans* infective larvae, which may easily be confused with other microfilaria species because of their morphological similarities. In the present study, we relied on a LAMP to detect *M. perstans* in the *Culicoides* as used by Poole et al. [17]. As an improvement in existing *M. perstans* diagnostic techniques mainly microscopy and PCR, Poole et al. [17] developed a LAMP assay that offered considerable advantages such as increased specificity, faster detection time, and requirement of less expensive equipment for performance. It also offers several alternate and easy ways of visualizing results as compared to PCR. With a limit of detection of 0.1 pg (equivalent to 1/1000th) fragment of an *M. perstans* microfilaria, and a specificity of 100%, it can detect *M. perstans* in the blood of infected patients and also identify *M. perstans* in infected *Culicoides* species [17]. New diagnostic tools with improved diagnostic capacity that are field-friendly and useful in resource-limited settings are needed to improve investigations of NTDs to achieve the Sustainable Development Goals.

In a defined geographical area, the *Culicoides* abundance and diversity strongly depend on the availability and type of breeding sites [10]. Communities in the Asante Akim North district on average recorded a higher species diversity and abundance than the Sene West district.

Afrisere and Abutantri recorded the highest *Culicoides* abundance, which could be attributed to the presence of favourable vegetation and breeding sites that enhances *Culicoides* larval development. The presence of plantations, livestock, and relatively thicker trees and bushes in these communities is known to provide suitable habitats for the vectors [15]. Bebuso in the Asante Akim North district recorded the fewest *Culicoides*. This could be attributed to the absence of livestock (specifically cattle, sheep, goats, and wild game), which serves as a main blood meal source, and their moist dung, which also provides a fertile breeding ground for some species. This is in line with the findings of Kameke et al. [11] who reported a very high abundance of *Culicoides* in livestock stables compared to regions farther from stables.

The biting patterns of anthropophilic species were determined by HLC. This is because trap collections are reported to be inaccurate in estimating the biting rate of *Culicoides* species [19]. HLC identified *C. grahamii, C. milnei*, and *C. inornatipennis*, which suggests these species are preferentially anthropophilic. *Culicoides grahamii* and *C. milnei*, being the most anthropophilic, accounted for 66% and 26% of the entire anthropophilic species, respectively. Wanji et al. [10], in Cameroon, also identified these three species, among others, as anthropophilic. In the present study, *C. inornatipennis* was the least anthropophilic species, which does not agree with earlier findings by Debrah et al. [12], who reported *C. inornatipennis* as the only anthropophilic species, and was also abundant in the Asante-Akyim North district. Over the years, climatic changes, evolving and emerging farming practices (such as the use of insecticides and weedicides), asnd urbanization may have adversely affected the abundance of *C. inornatipennis*. Notably, the most anthropophilic species (*C. grahamii* and *C. milnei*) were present in both districts. *Culicoides grahamii* and *C. milnei* exhibited similar biting patterns, with a peak biting time between the hours of 5 and 6 p.m., indicating that the time of maximum human-vector contact occurs around 5 to 6 p.m. Between the hours of 6 and 7 p.m., *C. milnei* showed the highest biting activity among all the anthropophilic species. This finding agrees with that of Wanji et al. [10] who reported that *C. milnei* is essentially a nocturnal species. Regarding the biting pattern of *C. grahamii*, our finding is in contrast to that of Hopkins [20] since he found this species to be only diurnal. This may be a result of differences in diurnal temperatures as these differences have been shown to influence periodicity or flight activity [21]. Increasing the time frame of human collections may provide a better overview of the biting patterns of the different *Culicoides* identified by HLC.

UV light traps collected approximately 95% (4557 out of 4810) of all the *Culicoides* recorded, and this included all six species identified, namely *C. inornatipennis*, *C. imicola*, *C. grahamii*, *C. milnei*, *C. neavei*, and *C. schultzei*. The high numbers collected using the light trap indicated that the UV light emitted by the trap serves as a great attractant to the midges as they not only follow any light source but also the kind of light emitted. Therefore, UV light traps are recommended for large-scale entomological surveys [21]. *Culicoides imicola* was the most abundant species in this study and a similar observation was made by Debrah et al. [12]. This may be due to the presence of livestock, the known preferred animal host for *C. imicola* [19] in most of the study communities. Despite the similarities in findings, the species diversity in the middle belt of Ghana as reported in the present study differs from the findings of Debrah et al. [12] who reported the presence of *Culicoides accraensis* and *C. fulvithorax*, which were not identified in this study. This is suggestive of either a drastic reduction in the abundance of these species due to the unfavourable climatic changes over the years or probably the unavailability of their preferred host(s).

Regarding seasonal variation in *Culicoides* abundance, there was a higher abundance of *Culicoides* in the rainy season, as similarly reported by Silva & Carvalho [22] and Debrah et al. [12], than the dry season. The excessive rainfall, which kept the soil and other plant matter moist, provided suitable conditions for *Culicoides* larval development and might have contributed to the higher abundance of the *Culicoides* species in the rainy season. The trend of higher *Culicoides* abundance in the rainy season was observed for all *Culicoides* species except *C. neavei*, which suggests that arid conditions enhance the growth of *C. neavei* pupal and larval stages [23]. Also, it is generally observed that there is a higher occurrence of bush burning in the dry season in Ghana. The destruction of vegetation covers and moist breeding sites might have contributed to the low abundance of the *Culicoides* species captured in the dry season in both districts.

## Conclusions

In conclusion, we have identified, for the first time to our knowledge, *Culicoides grahamii* as a potential vector for *M. perstans* in the middle belt of Ghana.

### Supplementary Information

**Additional file 1: Table S1.** Specific sequences of *Mansonella* *perstans *primer set. FIP (forward inner primer), BIP (backward inner primer), F3 (forward outer primer), B3 (backward outer primer), LF (forward loop), LB (backward loop), µM (micromole), H_2_O (water).

**Additional file 2: Table S2.** Preparation of 10× primer mix. FIP (forward inner primer), BIP (backward inner primer), F3 (forward outer primer), B3 (backward outer primer), LF (forward loop), LB (backward loop), µM (micromole), H_2_O (water).

**Additional file 3: Table S3.** Preparation of 20 µl reaction mix. Preparation of 10× primer mix. DNA (deoxyribonucleic acid), GuHCl (guanidine hydrochloride), µl (microliters).

## Data Availability

The datasets supporting the conclusions of this article are included within the article and in Tables 1 and 2, Figs. 1, 2, 3, and 4.

## Abbreviations

CDC: Centre for Disease Control and Prevention
L3: Third Larval Stage
LAMP: Loop-mediated isothermal amplification
MF: Microfilariae
HLC: Human landing catch
NTD: Neglected tropical disease
