# Supplementary material for: Diversity of Culicoides in the middle belt of Ghana with Implications on the transmission of Mansonella perstans; a molecular approach
Source: Parasit Vectors. 2024 Mar 12;17:123. doi: 10.1186/s13071-024-06179-8 (PMC10936074; doi:10.1186/s13071-024-06179-8)
Supplement: Supplementary file 1 — Additional file 1: Table S1. Specific sequences of Mansonella perstans primer set. FIP (forward inner primer), BIP (backward inner primer), F3 (forward outer primer), B3 (backward outer primer), LF (forward loop), LB (backward loop), µM (micromole), H2O (water). [file 13071_2024_6179_MOESM1_ESM.docx]

**Supplementary information**

**Additional file 1: Table S1** Specific sequences of *M. perstans* primer set. FIP (Forward Inner Primer), BIP (Backward Inner Primer), F3 (Forward Outer Primer), B3 (Backward Outer Primer), LF (Forward Loop), LB (Backward Loop), µM (micro-Molar), H2O (water).

| Primer | Sequence (5’-3’) |
| --- | --- |
| Mpe_FIP (F1c + F2) | TGTGAGCACATTTCAGTAAGT-GATGAAATCCACTAAATTCWC |
| Mpe_BIP (B1 + B2c) | GGATTCTTTCTAAAAGTTGAG-GATCGATTTCGTTAAAAACAGY |
| Mpe_F3 | ACAGTTGATTATTTGAAGGTGCTR |
| Mpe_B3 | AYAATGATTATTTYTAAAGAATC |
| Mpe_LF | AGACTTGATTACTGTTTGG |
| Mpe_LB | ACAATTTGGTAATCGCTTAAACTG |
