# Supplementary material for: Diversity of Culicoides in the middle belt of Ghana with Implications on the transmission of Mansonella perstans; a molecular approach
Source: Parasit Vectors. 2024 Mar 12;17:123. doi: 10.1186/s13071-024-06179-8 (PMC10936074; doi:10.1186/s13071-024-06179-8)
Supplement: Supplementary file 2 — Additional file 2: Table S2. Preparation of 10× primer mix. FIP (forward inner primer), BIP (backward inner primer), F3 (forward outer primer), B3 (backward outer primer), LF (forward loop), LB (backward loop), µM (micromole), H2O (water). [file 13071_2024_6179_MOESM2_ESM.docx]

**Additional file 2: Table S2** Preparation of 10X Primer mix. FIP (Forward Inner Primer), BIP (Backward Inner Primer), F3 (Forward Outer Primer), B3 (Backward Outer Primer), LF (Forward Loop), LB (Backward Loop), µM (micro-Molar), H2O (water).

| **Primer** | **1X Concentration (µM)** | **10X Concentration (µM)** | **µL of 100 µM primer stock** |
| --- | --- | --- | --- |
| 100 µM FIP | 1.6 | 16 | 16 |
| 100 µM F3 | 0.2 | 2 | 2 |
| 100 µM BIP | 1.6 | 16 | 16 |
| 100 µM B3 | 0.2 | 2 | 2 |
| 100 µM LF | 0.4 | 4 | 4 |
| 100 µM LB | 0.4 | 4 | 4 |
| H_2_O | ----- | ----- | 56 |
| Total Volume (µL) | ----- | ----- | 100 |
