# Supplementary material for: Diversity of Culicoides in the middle belt of Ghana with Implications on the transmission of Mansonella perstans; a molecular approach
Source: Parasit Vectors. 2024 Mar 12;17:123. doi: 10.1186/s13071-024-06179-8 (PMC10936074; doi:10.1186/s13071-024-06179-8)
Supplement: Supplementary file 3 — Additional file 3: Table S3. Preparation of 20 µl reaction mix. Preparation of 10× primer mix. DNA (deoxyribonucleic acid), GuHCl (guanidine hydrochloride), µl (microliters). [file 13071_2024_6179_MOESM3_ESM.docx]

**Additional file 3: Table S3** Preparation of 20 µL reaction mix. Preparation of 10X primer mix. H_2_O (water), DNA (Deoxyribonucleic Acid), GuHCl (Guanidine hydrochloride), µL (microliters).

|  | Plus, DNA | No DNA (non-template control) |
| --- | --- | --- |
| Warm start colorimetric LAMP 2X Master Mix – MI1800 | 10.0 µL | 10.0 µL |
| Primer Mix (10X) | 2.0 µL | 2.0 µL |
| 10X GuHCl | 2.0 µL | 2.0 µL |
| DNA | 2.0 µL | …. |
| H_2_O | 4.0 µL | 10.0 µL |
